# A Preliminary Study on the Role of Orexin A in Leydig Cell Steroidogenesis and Its Implications for Fertility in Alpacas (*Vicugna pacos*)

**DOI:** 10.3390/ani16040545

**Published:** 2026-02-10

**Authors:** Fabio Castagna, Roberto Bava, Stefano Ruga, Emilia Langella, Anna Costagliola, Renato Lombardi, Nicola Mirabella, Giovanna Liguori

**Affiliations:** 1 Department of Health Sciences, University of Catanzaro Magna Graecia, 88100 Catanzaro, Italy; fabiocastagna@unicz.it (F.C.); roberto.bava@unicz.it (R.B.); 2 School of Agricultural, Forestry, Food and Environmental Sciences (SAFE), University of Basilicata, 85100 Potenza, Italy; 3 Department of Veterinary Medicine and Animal Production, University of Naples Federico II, 80137 Naples, Italy; anna.costagliola@unina.it (A.C.); nicola.mirabella@unina.it (N.M.); 4 Local Health Authority (ASL), 71121 Foggia, Italy; renato.lombardi@aslfg.it (R.L.); giovanna.liguori@aslfg.it (G.L.)

**Keywords:** orexin A (OxA), orexin receptor 1 (OX1R), testis, reproductive health, steroidogenesis, alpaca

## Abstract

In Italy, the breeding of alpacas (*Vicugna pacos*) is on the rise, driven by the value of their precious wool and their docile temperament. This growth is, however, severely limited by the species’ reproductive inefficiency. This study aimed to investigate the specific role of the neuropeptide orexin A (OxA) and its receptor OX1R in modulating testicular steroidogenesis in the alpaca, testing the hypothesis that the OxA/OX1R pathway stimulates testosterone production. Using testicular tissue from two groups of adult male alpacas (five- and seven-year-olds, *n* = 6 per group), we combined immunohistochemistry with ex vivo functional assays. Immunohistochemical analysis confirmed the expression of both OxA and OX1R in Leydig cells. Functional studies on testicular fragments demonstrated that OxA enhanced testosterone synthesis via OX1R activation. Furthermore, OxA reduced estradiol levels by suppressing aromatase activity and counteracted the inhibitory effect of Müllerian inhibiting substance (MIS) on testosterone secretion. These results provide the first evidence that the peripheral OX1R pathway is functionally involved in promoting a testosterone-dominant steroidogenic milieu in alpaca testes. This preliminary study identifies the orexin system as a potential target for developing strategies to improve reproductive management in this species.

## Simple Summary

To support the sustainable growth of alpaca farming in Italy, hindered by the species’ reproductive challenges, this study examined a natural signaling system involving orexin A (OxA). Investigations using testicular tissue revealed that this system is active in the hormone-producing cells of alpacas. The research demonstrated that OxA effectively increases testosterone levels and reduces the action of other substances that inhibit this key fertility hormone. This evidence identifies the orexin pathway as an important regulator of testicular function, offering a scientific foundation for future approaches to enhance reproductive management in alpaca breeding.

## 1. Introduction

The alpaca (*Vicugna pacos*) is a South American camelid that has long been known for its superior fiber quality [1]. Alpaca breeding has spread far beyond the Andes, even to Italy [2,3]. The opening of alpaca farms in Italy follows the growing trend in the number of registered alpaca farms established in areas such as Europe, North America, and Australia [4,5]. This is also due to the calm and gentle nature of this species [6], making them suitable for Animal-Assisted Interventions (AAI) [7,8]. However, the development of the sector is severely limited by the reproductive inefficiency of the species [9,10,11].

This challenge is rooted in the intrinsic biology of the species: (1) ovulation is induced in females of the species [11]; (2) males suffer from delayed separation of the foreskin from the glans, which hinders penile extrusion; this condition results in a late functional sexual maturity, typically only at around 2.5–3 years of age, which is considerably later than in other common farm ruminants [11,12]; (3) sperm production is poor, resulting in a small ejaculate volume [13,14]; (4) a large amount of spermatozoa present morphological abnormalities [15]. Taken together, these conditions pose obstacles to assisted reproductive interventions and other integrated clinical approaches [16,17]. Nutritional approaches that include quality dietary supplementation lead to increased reproductive performance [18], which, however, cannot compensate for the anatomical and physiological limitations listed above.

Endogenous regulators of testicular function could represent important targets for research efforts to address this issue. In this regard, the orexin (OX) system is gaining increasing interest. Mediators of this system, orexin A (OxA) and orexin B (OxB), interact with G protein-coupled receptors, orexin 1 receptor (OX1R) and orexin 2 receptor (OX2R) [19]. The neuropeptide orexin, secreted in the hypothalamus, is a key regulator of central processes such as the sleep–wake cycle and appetite, thus governing energy homeostasis. Interestingly, these receptors have a ubiquitous distribution: they are not only present at the central level, but can also be found in the periphery and specifically, at the level of the gastrointestinal tract, at the level of the pancreas, the adrenal gland, the kidney, and even at the level of the capillary endothelium [20,21,22,23,24]. Equally important and functional for this study is the localisation of receptors in the testicles, as evidenced in various species, including rats, dogs, and, significantly, alpacas [19,25,26]. Such a widespread distribution makes it natural to think that there is a series of unclear physiological functions expressed at the peripheral level.

Preliminary studies describe a role for orexin in modulating steroidogenesis [27], by promoting testosterone production in Leydig cells [25,26,28]. Nevertheless, the effects that the binding between the neuropeptide and its receptor produces at the testicular level remain unclear. The present study was, therefore, designed to test the hypothesis that the OxA/OX1R pathway stimulates testosterone production and modulates the local hormonal milieu in alpaca testes. To this end, the function of the OxA/OX1R link in testicular steroidogenesis was investigated using immunohistochemical techniques combined with ex vivo functional tests. Specifically, this work aimed to determine whether OxA can stimulate testosterone production, modulate aromatase activity, and counteract the inhibitory signals of Müllerian inhibiting substance (MIS). A better understanding of these mechanisms could provide foundational knowledge for future strategies aimed at improving reproductive management in this species.

## 2. Materials and Methods

### 2.1. Animal Handling and Experimental Design

This study was conducted at the Experimental Physiology Laboratory of the Department of Veterinary Medicine and Animal Production, University of Naples Federico II, Italy. The animals enrolled came from an accredited Italian farm located in the Campania region. The subjects were in optimal nutritional conditions, as assessed by a body condition score (BCS) of 3.0–3.5 on a 5-point scale. Mean body weight was 68.5 ± 3.2 kg (mean ± SEM). The alpacas were raised semi-free range, with access to pasture during the day and shelter at night. 

Their diet consisted of mixed grass hay ad libitum, supplemented with 400 g/head/day of a commercial concentrate pellet formulated for camelids, and mineral salt blocks. Water was always available. Specifically, two groups of sexually mature male alpacas were enrolled (*n* = 6 per group, half 5 years of age and the other half 7 years of age). These subjects had their testes removed via orchiectomy, performed under local anesthesia, according to standard procedures established by Fowler (1989) [29]. Written informed consent has been obtained from the owner of the animals involved in this study. The animal study protocol was approved by the Institutional Review Board (or Ethics Committee) of the Committee for the Care and Ethical Use of Animals of the University of Naples Federico II, Department of Veterinary Medicine and Animal Production (protocol code 0024889, approved on 12 March 2018).

### 2.2. Antibodies and Chemicals

Key reagents were obtained as follows: peptides and chemicals from Phoenix Pharmaceuticals Inc. (OxA, cat. 003-30; Karlsruhe, Germany) and Sigma-Aldrich/Sigma Chemical Co. (SB-408124, LH [L5269], BSA; St. Louis, MO, USA). Primary antibodies were from R&D Systems (monoclonal anti-OxA, MAB763; Abingdon, UK) and Millipore (polyclonal anti-OX1R, ab3092; Burlington, MA, USA). The synthetic peptide corresponding to the anti-OxA antibody, used for pre-adsorption controls, was supplied by Tocris Bioscience (Bristol, UK). Secondary antibodies, the avidin-biotin complex (ABC) kit (PK-6105), and the DAB substrate were all sourced from Vector Laboratories (Burlingame, CA, USA). Hormone quantification was performed using a testosterone enzyme immunoassay (EIA) kit from Adaltis (Bologna, Italy) and an estradiol enzyme-linked immunosorbent assay (ELISA) kit from Diametra (cat. DKO003; Perugia, Italy).

### 2.3. Immunohistochemistry

Testicular tissue samples were collected immediately after orchiectomy and fixed by immersion in Bouin’s solution for 24 h. Following fixation, samples were paraffin-embedded, and 7 μm-thick sections were prepared. After deparaffinization and rehydration, antigen retrieval was performed using citric acid buffer (pH 6.0). Immunostaining was carried out using the avidin-biotin-peroxidase complex (ABC) method (Vectastain ABC Kit, PK-6105, Vector Laboratories, Burlingame, CA, USA). Sections were incubated overnight at 4 °C with primary antibodies diluted in PBS-BSA 1% under the following conditions: mouse anti-OxA monoclonal antibody (MAB763, R&D Systems) at a 1:150 dilution and rabbit anti-OX1R polyclonal antibody (ab3092, Millipore) at a 1:200 dilution. Subsequently, sections were incubated with the appropriate biotinylated secondary antibodies (Goat anti-mouse BA-9200 or Goat anti-rabbit BA-1000, Vector Laboratories) for 1 h at room temperature, followed by the ABC for 30 min.

The immunoreaction was visualized using 3,3′-diaminobenzidine (DAB) as a chromogen, and sections were counterstained with hematoxylin. For negative controls, the primary antibody was either omitted or pre-adsorbed with a five-fold molar excess of the corresponding synthetic peptide (Tocris Bioscience) for 2 h at room temperature before application. No specific immunostaining was observed in these controls. Immunohistochemical assessment was performed by two independent observers blinded to the experimental groups. For each animal, three non-serial sections were analyzed. From each section, 10 random fields of the interstitial compartment were captured at 40× magnification using a Leica DM6 B light microscope equipped with an SFC7000T digital (Leica Microsystems, Wetzlar, Germany )imaging system. All OxA- and OX1R-immunopositive Leydig cells within these fields were manually counted. The area of the interstitial compartment in each field was measured using ImageJ software (version 1.52a of 2018, NIH, Madison, WI, USA). Data are expressed as the number of immunopositive cells per square millimeter of interstitial area (cells/mm^2^) and presented as mean ± SEM for each age group.

### 2.4. In Vitro Assessment of Testicular Steroidogenesis: Testosterone Secretion and Aromatase Activity

Based on previous work by Liguori et al. [25,26], 2018, two complementary in vitro methodologies were employed to investigate testicular steroidogenesis. Testicular tissues were decapsulated, sectioned into uniform fragments (~250 mg), and incubated in Krebs–Ringer bicarbonate buffer supplemented with 10 mM glucose, 0.1 mM bacitracin, 0.1 mM ascorbic acid, and 0.1% (*w*/*v*) bovine serum albumin, under controlled conditions (37 °C, 95% O_2_/5% CO_2_, agitation at 60 cycles/min). For testosterone secretion assays, tissues were preincubated for 60 min and then exposed to various treatments. These included single treatments with orexin A (OxA; 100 nM), the OX1R antagonist SB-408124 (10 µM), or Müllerian inhibitory substance (MIS; 100 ng/mL), as well as simultaneous co-treatments (OxA + SB-408124). 

To specifically investigate the temporal interaction between OxA and MIS, a sequential treatment protocol was adopted [25]. In this protocol: one group of fragments was first exposed to OxA (100 nM) for 6 h, after which MIS (100 ng/mL) was added to the medium and the incubation continued for an additional 6 h (OxA → MIS group, total 12 h); a separate group was first exposed to MIS (100 ng/mL) for 6 h, followed by the addition of OxA (100 nM) for the subsequent 6 h (MIS → OxA group, total 12 h).

Luteinizing hormone (LH) was used as a viability control in all setups. Testosterone was extracted with ether and quantified using an enzyme-linked immunosorbent assay, which had a sensitivity of 6 pg per well and a recovery of approximately 80%, and further investigations were conducted to quantify 17β-estradiol production and aromatase (ARO) activity. The enzymatic conversion of testosterone to estradiol was measured by exposing tissue fragments to OxA and SB-408124 in the presence of testosterone and NADPH after a 12-h incubation period. Estradiol quantification was performed by ELISA, with a sensitivity of 4 pg per well and a recovery of approximately 85%. All tests were performed in triplicate, and procedures were standardized with samples normalized for tissue weight and incubation duration. These data are summarized in Table 1.

### 2.5. Statistical Analysis

GraphPad Prism 10 (GraphPad Software, Inc., La Jolla, CA, USA) was used for all statistical analyses. The data were normalized to grams of tissue and analyzed in triplicate. To assess the normality of the distribution, the Shapiro–Wilk test was performed. A Student’s *t*-test was then conducted to compare the groups, with statistical significance considered for *p*-values below 0.05. The results are presented as the standard error of the mean (SEM).

## 3. Results

### 3.1. Immunolocalization and Quantification of Orexin A and OX1R

**Figure 1:**
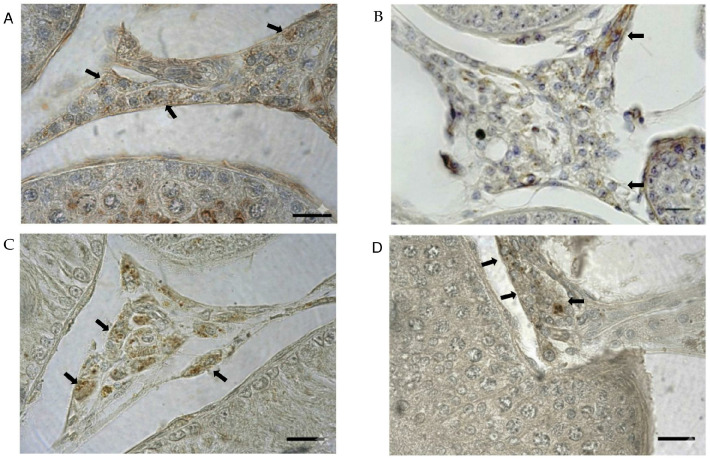
Immunohistochemical localization of Orexin A (OxA) and Orexin-1 receptor (OX1R) in the alpaca testis. Leydig cells immunopositive for OxA are shown in 7-year-old (**A**) and 5-year-old (**B**) subjects, while Leydig cells immunopositive for OX1R are illustrated in 7-year-old (**C**) and 5-year-old (**D**) subjects. Immunostaining was observed in Leydig cells displaying condensed granular material and immunoreactivity for OX1R and OxA (arrows). Immunohistochemistry was carried out using the avidin–biotin method. Scale bars represent 20 µm.

Leydig cells of testicular tissue showed positive immunoreactivity for OxA and OX1R. The response had a cytoplasmic location and a granular appearance. Abundant Leydig cells were positive (Figure 1).

These cells were grouped in small clusters, comprising variably pigmented parts, and had a round or oval shape. Microscopic analysis of tissue slices revealed roughly 20 different Leydig cell clusters, each consisting of 15–25 cells.

### 3.2. Orexin A Stimulates Testosterone Production via OX1R

**Figure 2:**
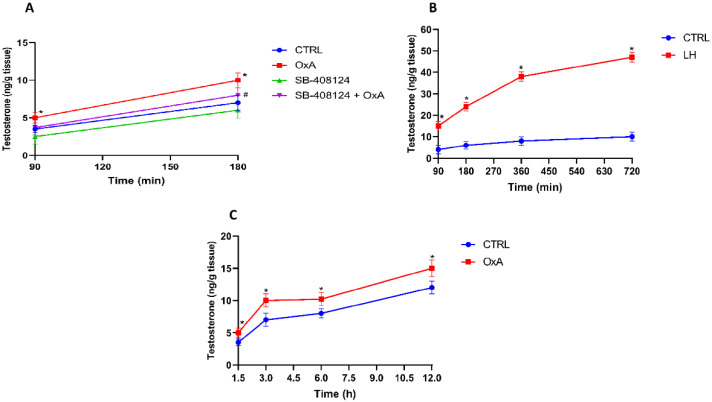
(**A**) Effect of OxA and SB-408124 on Testosterone Levels in Leydig Cells. * *p* < 0.05 vs. CTRL, # *p* < 0.05 vs. OxA 180 min; (**B**) Time-Dependent Effect of LH on Testosterone. * *p* < 0.05 vs. CTRL; (**C**) Time-Course of Testosterone Increase Induced by OxA. * *p* < 0.05 vs. CTRL.

After 90 min, OxA induced a statistically significant increase in testosterone levels compared to CTRL (*p* < 0.05), and this difference remained significant at 180 min (*p* < 0.05). The OX1R antagonist SB-408124 alone had no significant effect on testosterone levels compared to CTRL. However, T levels in the SB-408124 + OxA were significantly lower than in the OxA group at 180 min (*p* < 0.05) (Figure 2A).

LH administration significantly increased T levels compared to CTRL across all time points from 90 to 720 min (*p* < 0.05) (Figure 2B).

OxA significantly increased T levels compared to CTRL from 1.5 h to 12 h (*p* < 0.05) (Figure 2C).

### 3.3. Orexin A Counteracts the Inhibitory Effect of MIS on Testosterone

**Figure 3:**
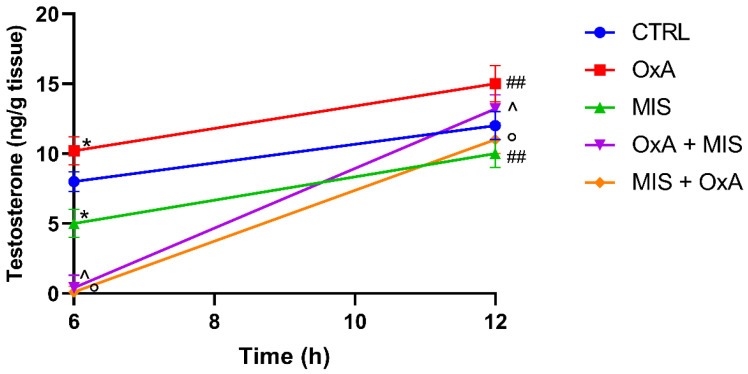
Effect of OxA and MIS on Testosterone Levels Over Time. * *p* < 0.05 vs. CTRL, ## *p* < 0.01 vs. CTRL, ^ *p* < 0.05 vs. OxA, ° *p* < 0.05 vs. MIS. For sequential treatments (OxA → MIS and MIS → OxA), testosterone levels measured after the first 6 h incubation were set as time 0.

After 6 h, OxA significantly increased T levels compared to CTRL (*p* < 0.05), and this difference remained significant at 12 h (*p* < 0.01). MIS alone significantly reduced T levels at 6 h (*p* < 0.05) and 12 h (*p* < 0.01) compared to CTRL. In sequential treatments, when OxA was added first, followed by MIS (OxA → MIS), testosterone levels were significantly lower compared to OxA alone at both 6 h (*p* < 0.05) and 12 h (*p* < 0.05). Similarly, when MIS was added first, followed by OxA (MIS → OxA), testosterone levels were also significantly lower compared to OxA alone at both time points (*p* < 0.05) (Figure 3).

### 3.4. Orexin A Suppresses Estradiol Synthesis and Aromatase Activity via OX1R

**Figure 4:**
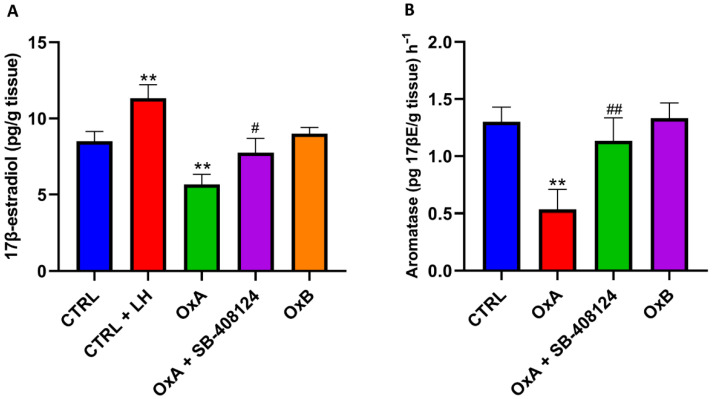
(**A**) Effect of OxA, LH, and SB-408124 on 17β-Estradiol Levels. ** *p* < 0.01 vs. CTRL, # *p* < 0.05 vs. OxA; (**B**) Effect of OxA, OxB, and SB-408124 on Aromatase Expression. ** *p* < 0.01 vs. CTRL, ## *p* < 0.01 vs. OxA.

17β-estradiol levels were significantly increased in the CTRL + LH group compared to CTRL (*p* < 0.01). In contrast, OxA significantly reduced 17β-estradiol levels compared to CTRL (*p* < 0.01). This reduction was partially reversed by SB-408124, with a significant increase compared to OxA alone (*p* < 0.05). OxB did not induce significant changes in estradiol levels compared to control (Figure 4A).

OxA significantly reduced aromatase levels compared to CTRL (*p* < 0.01). This reduction was reversed by SB-408124, with significantly increased levels compared to OxA (*p* < 0.01). OxB showed no significant differences in aromatase levels compared to CTRL (Figure 4B).

### 3.5. Expression and Quantification of Orexin A and OX1R in Leydig Cells

**Figure 5:**
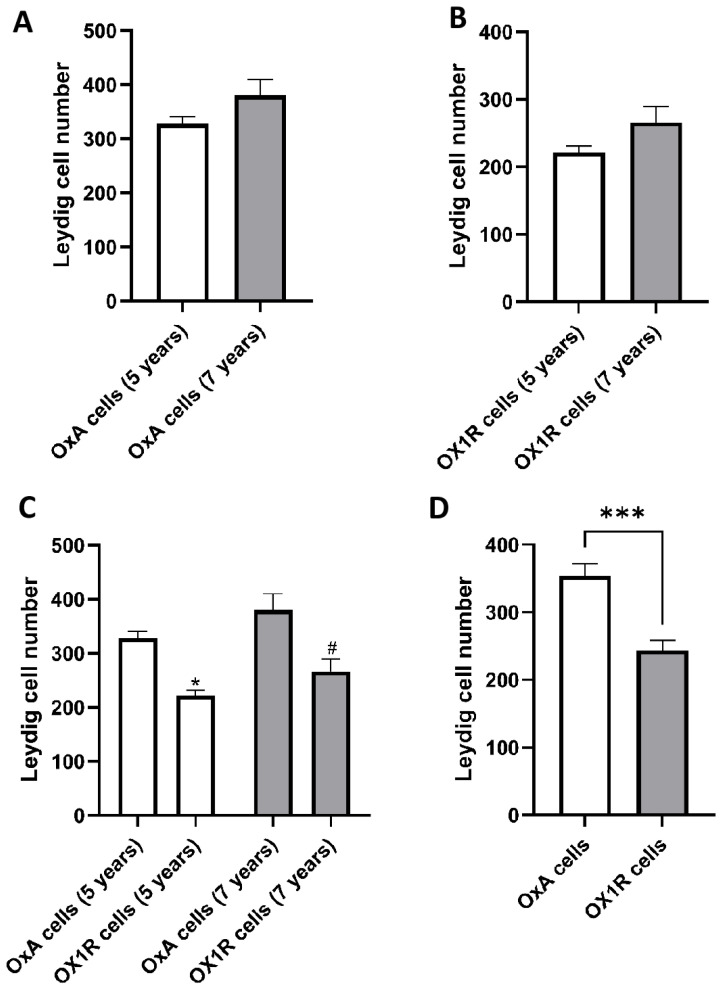
(**A**) Quantification of OxA-Positive Leydig Cells in 5- and 7-Year-Old Alpacas; (**B**) Quantification of OX1R-Positive Leydig Cells in 5- and 7-Year-Old Alpacas; (**C**) Comparison of OxA- and OX1R-Positive Leydig Cells in Alpacas by Age. * *p* < 0.05 vs. OxA cells (5 years), # *p* < 0.05 vs. OxA cells (7 years); (**D**) Total Number of OxA- and OX1R-Positive Leydig Cells in Alpaca Testis. *** *p* < 0.001.

No statistically significant differences were observed in the number of OxA-positive Leydig cells between 5- and 7-year-old alpacas (Figure 5A). No statistically significant differences were observed in the number of Ox1R-positive Leydig cells between 5- and 7-year-old alpacas (Figure 5B). In both age groups, the number of OX1R-positive cells was significantly lower than the number of OxA-positive cells (*p* < 0.05) (Figure 5C). The total number of OX1R-immunopositive Leydig cells was significantly lower than the number of OxA-positive cells (*p* < 0.001) (Figure 5D).

## 4. Discussion

Orexins, acting via their receptors OX1R and OX2R, constitute a conserved and flexible regulatory network in the male gonad, integrating endocrine, paracrine, and metabolic signals to modulate steroidogenesis, spermatogenesis, and germ cell survival. Comparative studies across humans, rodents, dogs, ruminants, and boars reveal both conserved principles and species-specific adaptations, suggesting evolutionary tuning of orexin signaling to reproductive strategy, metabolic status, and gonadal architecture [30,31,32,33,34,35].

In humans, OX1R and OX2R localize to Leydig cells, Sertoli cells, and testicular peritubular myoid cells, with additional expression in accessory structures such as the epididymis, seminal vesicles, and penis [32]. This distribution parallels rodents, where Leydig and Sertoli cells are primary targets for OxA, though OX1R dominates in rats, while murine OX2R is more relevant in both interstitial and tubular compartments [30,35,36,37].

Functional studies indicate that OxA stimulates testosterone production in Leydig cells, potentially establishing a feedback loop in which testosterone regulates receptor expression while orexin binding enhances steroidogenesis [37,38,39]. OXB, in contrast, generally lacks direct steroidogenic activity in rats, but in mice, OXB/OX2R signaling appears to fine-tune germ cell proliferation and Leydig cell development, as OX2R knockdown increases T and germ cell proliferation, suggesting a modulatory or suppressive basal role [34,35].

Rodents provide compelling evidence for orexin-mediated metabolic support of spermatogenesis. OxA/OX1R signaling in Sertoli cells maintains glucose uptake via GLUT3 and GLUT8, supporting lactate production that fuels germ cells [30,40,41]. Antagonism of OX1R reduces glucose and lactate levels, increases pro-apoptotic markers (p53, Bax, caspase-3), decreases anti-apoptotic Bcl-2, and reduces PCNA-positive germ cells, culminating in germ cell apoptosis and impaired proliferation [30,42,43]. While metabolic data are less extensive in humans and dogs, similar Sertoli cell receptor localization suggests that orexin-mediated support of germ cell metabolism may be conserved, albeit with species-specific intensity.

Our present findings in male alpacas extend these concepts and underscore both conserved and species-specific features. Immunohistochemical analysis confirmed the presence of OxA and OX1R in interstitial Leydig cells, showing cytoplasmic localization and clustered distribution reminiscent of murine and canine models [25,26,39]. Expression was stable across two adult age cohorts, likely reflecting sexual maturity and peak Leydig cell function. Quantitatively, the number of OxA-positive Leydig cells significantly exceeded OX1R-positive cells (*p* < 0.05), suggesting that receptor availability rather than ligand concentration constrains pathway activation in vivo.

Several limitations of this study must be acknowledged. First, the ex vivo approach, while controlled, excludes integral physiological influences such as neuronal input, vascular dynamics, and pulsatile hormonal stimuli present in vivo. Furthermore, the OxA concentrations used, while effective, may not precisely mirror physiological levels. Another limitation is the exclusive focus on OX1R, leaving the potential role of OX2R unexplored, as it is also expressed in the testes of some species. Our sample size (*n* = 6 per group), though statistically adequate for the primary endpoints, may limit the detection of subtler age-related or individual variations in protein expression. Finally, the lack of correlation with semen parameters or fertility outcomes, due to the study design, means that while we demonstrate a clear endocrine effect, a direct link to improved reproductive performance remains to be established. Therefore, future in vivo studies are essential to translate these findings into practical applications.

Time-dependent T increases were markedly inhibited by the selective OX1R antagonist SB-408124, confirming receptor specificity, consistent with rodent studies showing orexin-induced activation of steroidogenic enzymes and mitochondrial activity [25,37]. OxA also counteracted Müllerian inhibiting substance (MIS)–mediated suppression of testosterone, establishing a local counter-regulatory mechanism to preserve androgen output [31,33,34]. Beyond enhancing androgens, OxA decreased 17β-estradiol and suppressed aromatase activity [28], shifting the steroidogenic balance toward a more androgenic profile—a dual modulatory action also observed in the testes of other species. For example, in mice, OX1R antagonism reduces expression of SF1, StAR, P450scc, and 17β-HSD, lowering T levels, while paradoxically increasing 3β-HSD and P450arom expression and estradiol (E2) levels [44]. Hence, orexin signaling integrates steroidogenic, metabolic, and antioxidant pathways to sustain testicular function.

While OX1R dominates OxA-mediated steroidogenesis, OX2R expression in multiple mammalian testes suggests that a portion of OxA signaling may be mediated via OX2R on distinct cell populations, contributing to germ cell proliferation and steroidogenic regulation [34,35]. In ruminants, orexin signaling appears more adapted to environmental and metabolic cues rather than direct steroidogenic stimulation. In mature rams and bucks, testicular size and seminiferous tubule number are influenced by food intake, affecting spermatogenic efficiency without necessarily altering T production or sexual behavior [45]. OX1R predominates in Leydig cells and adrenal glands, whereas OX2R expression is minimal in the sheep pineal gland and adrenal, in contrast to rodents, indicating species-specific divergence in orexin-mediated reproductive regulation [46].

Stage-specific and pathological variations are notable. In rodents, OXA is expressed in Leydig cells, Sertoli cells, spermatocytes, and spermatids in a germ cell cycle-dependent manner [37,47], whereas in mice, OxB/OX2R spans from spermatogonia to elongating spermatids, with OxA/OX1R additionally regulating Sertoli cell metabolism [35,36]. In dogs, OxA/OX1R stimulates T, whereas OXB/OX2R may act paracrinely without steroidogenic effects; in cryptorchid dogs, OxB/OX2R distribution is restricted to Leydig and Sertoli cells, correlating with impaired spermatogenesis [26,31]. In alpacas, the predominance of OxA-positive Leydig cells over OX1R-positive cells may reflect an analogous paracrine amplification mechanism, with potential implications for optimizing androgen output in populations with suboptimal steroidogenesis.

Collectively, these comparative data suggest that OxA/OX1R signaling is the principal conserved mechanism for stimulating testosterone production, supporting Sertoli cell metabolism, and regulating germ cell survival. Rodents exemplify metabolic and apoptotic regulation, humans and dogs highlight endocrine and paracrine modulation, and alpacas provide a unique model in which OxA/OX1R may compensate for reproductive inefficiencies, including suboptimal steroidogenesis.

Finally, evolutionary divergence likely explains species-specific receptor patterns. The presence of both OX1R and OX2R in human and rodent Leydig cells, versus OX1R predominance in ruminants and boars, indicates a flexible orexin system capable of integrating hypothalamic, nutritional, and photoperiodic signals to adaptively regulate male fertility.

Alpacas may exemplify the translational relevance of this system, where targeting OxA/OX1R may provide novel strategies to enhance steroidogenesis and reproductive performance. Future studies should incorporate in vivo models and evaluate sperm parameters, including motility, morphology, and maturation, to link Leydig cell endocrine function with gamete quality. Investigations into OX2R signaling, ligand–receptor interactions, and pharmacological modulation with peripherally restricted OX1R agonists will be essential for translational applications, minimizing potential central nervous system effects. Collectively, these findings provide a comprehensive mechanistic framework for the Orexin–testis axis in alpacas and highlight its potential as a target for interventions aimed at improving steroidogenesis and reproductive efficiency.

## 5. Conclusions

This study demonstrates that the OxA/OX1R system is functionally active in the alpaca testis. OxA and OX1R are co-expressed in Leydig cells, with OxA present at higher levels, and OxA acts as a potent stimulator of testosterone synthesis via specific OX1R activation. Additionally, OxA counteracts MIS-induced suppression of steroidogenesis and reduces aromatase activity, promoting an androgenic testicular environment. These results establish the OxA/OX1R axis as an important local regulator of steroidogenesis in alpacas and provide a foundation for future research aimed at exploring targeted interventions, such as selective OX1R modulation, to enhance reproductive efficiency in this species.

## Figures and Tables

**Table 1:** Comparative Overview of In Vitro Steroidogenesis Assays.

Parameter	Testosterone Secretion Assay	17β-Estradiol Production & Aromatase Activity Assay
Tissue Preparation	Decapsulated, sliced (~250 mg)	Same
Incubation Buffer	Krebs–Ringer bicarbonate + supplements	Same
Preincubation Time	60 min	60 min
Treatment Conditions	OxA, SB-408124, MIS (alone or combined), LH	OxA, SB-408124, LH; followed by T + NADPH for ARO assay
Incubation Duration	90 min to 180 min	12 h + additional incubation with T + NADPH
Steroid Extraction	Ethyl ether extraction	Ethyl ether extraction
Quantification Method	Enzyme immunoassay (EIA); sensitivity 6 pg	ELISA; sensitivity 4 pg
Recovery Efficiency	~80%	~85%
Normalization	Per gram tissue	Per gram tissue and time (pg/g/h)

## Data Availability

Data are available upon request to corresponding author.
